# Mixed organic acids improve nutrients digestibility, volatile fatty acids composition and intestinal microbiota in growing-finishing pigs fed high-fiber diet

**DOI:** 10.5713/ajas.18.0517

**Published:** 2018-10-29

**Authors:** Miao Li, Shenfei Long, Qianqian Wang, Lianhua Zhang, Jiangxu Hu, Jie Yang, Zhibin Cheng, Xiangshu Piao

**Affiliations:** 1 State Key Laboratory of Animal Nutrition, College of Animal Science and Technology, China Agricultural University, Beijing 100193, China; 2 Yunnan Kuaidaduo Animal Husbandry Technology Co., LTD, Yuxi 653100, China; 3 College of Animal Science and Technology, Yunnan Agricultural University, Kunming 650000, China

**Keywords:** Growing-finishing Pigs, Microbiota, Nutrients Digestibility, Organic Acids, Volatile Fatty Acids, Wheat Bran

## Abstract

**Objective:**

The objective of this study was to investigate effects of mixed organic acids (MOA) on nutrient digestibility, volatile fatty acids composition and intestinal microbiota in growing-finishing pigs fed high wheat bran diet.

**Methods:**

Six crossbred barrows (Duroc×Landrace×Yorkshire), with an average body weight 78.8±4.21 kg, fitted with T-cannulas at the distal ileum, were allotted to a double 3×3 Latin square design with 3 periods and 3 diets. Each period consisted of a 5-d adjustment period followed by a 2-d total collection of feces and then a 2-d collection of ileal digesta. The dietary treatments included a corn-soybean-wheat bran basal diet (CTR), mixed organic acid 1 diet (MOA1; CTR+3,000 mg/kg OA1), mixed organic acid 2 diet (MOA2; CTR+2,000 mg/kg OA2).

**Results:**

Pigs fed MOA (MOA1 or MOA2) showed improved (p<0.05) apparent total tract digestibility (ATTD) of gross energy, dry matter and organic matter, and pigs fed MOA2 had increased (p<0.05) ATTD of neutral detergent fiber compared to CTR. Dietary MOA supplementation decreased (p<0.05) pH value, and improved (p<0.01) concentrations of lactic acid and total volatile fatty acids (TVFA) in ileum compared to CTR. Pigs fed MOA showed higher (p<0.05) concentration of acetic acid, and lower (p<0.05) content of formic acid in feces compared to CTR. Pigs fed MOA1 had increased (p<0.05) concentration of TVFA and butyric acid in feces. Pigs fed MOA1 showed higher concentration of *Lactobacillus* and lower concentration of *Escherichia* in feces compared to CTR.

**Conclusion:**

Dietary supplementation of MOA 1 or 2 could improve nutrients digestibility, TVFA concentration and intestinal flora in growing-finishing pigs fed high fiber diet.

## INTRODUCTION

Due to their improving animal growth performance and altering the proportions of bacteria in the microbiome [1], the use of antibiotic growth promoters (AGP) to improve animal performance and health has been a usual practice during the last 50 years. But a full prohibition on the use of AGP in-feed came into effect in the European Union in 2006. Antibiotic consumption in animal feed contributes to the problems of antibiotic resistance, which raises worldwide concern that antimicrobial resistance has made drugs used to treat human disease less effective [2]. Moreover, excessive antibiotics give raise to residues in animal products and environment. Thus, searching for reliable replacements of antibiotic in diets fed to pigs has increased [3]. Organic acids (OA), as a alternative to antibiotics, have been widely applied in all phases of pig production.

Dietary acidification with OA has been shown to improve the average daily gain (ADG) and feed efficiency in weaned piglets and growing-finishing pigs [4,5]. Dietary OA supplementation improvement of apparent total tract digestibility (ATTD) of gross energy (GE), crude protein (CP), calcium, and phosphorus has been documented in previous studies in short chain fatty acid (SCFA)-fed and medium chain fatty acids (MCFA)-fed piglets [6]. It is generally considered that dietary OA could stimulate pancreatic secretions, such as trypsin, and also OA could serve as substrates in intermediary metabolism [7]. Besides, several studies have demonstrated that dietary OA improve the apparent ileal digestibility (AID) of protein and amino acids in growing-finishing pigs [8], which was associated with the lowering of gastric pH and increasing activity of proteolytic enzymes. The antibacterial activity of OA is dependent on their effects on lowering the pH of the digesta. Besides, OA could pass into bacterial cells and once inside the microorganisms cell change from undissociated to dissociated form and suppress cell enzymes (decarboxylases and catalases) and nutrient transport systems [9]. Meanwhile, dietary OA can produce a more favorable environment for *Lactobacillus*, which is beneficial for growth and health of animals [10]. The combination of OA (fumaric and lactic acid) and MCFA (caprylic acid and capric acid) resulted in decrease of the intestinal pH and reduction of *Escherichia coli* (*E. coli*) pathogenic genes [11]. The previous result in our group also indicates piglets fed with diets containing OA treated corn-soybean meal basal diet shows improved growth performance and nutrient digestibility [12,13].

Due to the high content of anti-nutritive factors, mainly non-starch polysaccharides (NSP), the use of wheat bran as feed material in pig nutrition is limited. Previous research showed that wheat bran has no effect on ADG and average daily feed intake [14]. An increased level of wheat bran in the diet decreased the ATTD of GE, but did not affect the ATTD of CP for growing-finishing pigs [15]. Besides, diet containing a high level fiber will influence the transit time with a reduction in the upper and increase in the lower digestive tract, therefore, increases the growth of microbial in the gastrointestinal tract. On the other hand, it will create the possibility for the proliferation of pathogenic bacteria such as *E. coli*, which negatively affects microbiological status [16,17]. Studies showed OA might regulate monogastric intestinal microbial flora and it is expected that OA might help to improve the utilization of dietary fiber in pigs. Previous research indicated that the combination of organic acids and MCFA had positive effects on the digestibility of nutrients as well as on growth performance of growing-finishing pig fed corn-soybean diet [5,8]. However, there are few studies focused on how mixed organic acid (MOA) used in diets with high fiber levels influence the nutrient digestibility and microbial metabolite concentrations in the ileum.

Therefore, the objectives of the current research were to determine effects of two MOAs on nutrients digestibility, ileal and fecal volatile fatty acids (VFAs) and microbiota on growing-finishing pig fed high wheat bran diet. We hypothesize that MOAs could improve the nutrients digestibility by lowering the intestinal pH and stimulating pancreatic secretions. The MOAs could increase the carbohydrate fermentation in large intestine and increase the concentration of total volatile fatty acids (TVFA), and improve the intestinal microbial environment.

## MATERIALS AND METHODS

All the procedures used in the following experiment were reviewed and approved by the Institutional Animal Care and Use Committee of China Agricultural University (Beijing, China, CAU20170902-2). This experiment was conducted at the Animal Experiment Center of Ministry of Agriculture Feed Industry Centre.

### Organic acids

The two commercial MOA products used in our research were produced in the Netherlands (Nutreco N.V., Amsterdam, Netherlands). MOA1 is a synergetic compound of free and buffered SCFAs (mainly formic acid, acetic acid, and propionic acid) combined with a small percentage of MCFA (mainly C8:0 and C10:0). MOA2 is a synergetic compound of a phenolic blend, mainly contain monounsaturated fatty acids and MCFA (mainly C10:0 and C12:0), target release butyrate and sorbic acid. The carrier for MOA1 and MOA2 is silica.

### Experimental design and diets

Six crossbred barrows (Duroc×Landrace×Yorkshire), with an average body weight 78.8±4.21 kg, were allotted to a double 3×3 Latin square design with 3 periods and 3 diets. Pigs were surgically fitted with a T-cannula in the distal ileum using procedures adapted from Stein et al [18]. Each period consisted of a 5-d adjustment period followed by a 2-d total collection of feces and then a 2-d collection of ileal digesta. The dietary treatments included a corn-soybean-wheat bran basal diet (CTR), mixed organic acid 1 diet (MOA1; CTR+3,000 mg/kg MOA1), and mixed organic acid 2 diet (MOA2; CTR+2,000 mg/kg MOA2). Vitamins and minerals were supplemented to meet or exceed the estimated nutrient requirements for pigs as recommended by the NRC [19]. Chromic oxide (2,500 mg/kg) was included in all diets as an inert marker. The ingredients composition of the experimental diets is shown in Table 1.

Pigs were housed in individual pens in an environmentally controlled room (22°C±2°C). A feeder and a nipple drinker were installed in each pen. Body weight was recorded at the beginning of the experiment. Feed allowance was equivalent to 40 g/kg of body weight and divided into 2 equal meals fed at 08:00 and 17:00 h each day. Water was available at all times throughout the experiment periods.

### Sample collection

Each period lasted 9 days. Feces were collected via grab sampling in the morning of d 6 and 7 in each period. The fecal samples were stored at −20°C quickly after collection. At the end of the experiment, feces were dried at 65°C for 72 h. Samples were ground through a 1mm screen after drying and then were stored at −20°C until used for chemical analysis. Ileal digesta was collected continuously for 12 h from 08:00 to 20:00 h on d 8 and 9 according to procedures described by Zeng et al [20]. In brief, a 200 mL plastic bag was attached to the open cannula using a cable tie. Bags were removed whenever they were filled with digesta or at least every 30 min and stored at −20°C to prevent bacterial degradation of the amino acids in the digesta.

### Chemical analyses

Feed and fecal samples were analyzed for CP, dry matter (DM), ash, neutral detergent fiber (NDF), and acid detergent fiber (ADF). Ileal digesta samples were analyzed for CP, DM, and ash according to the methods of AOAC [21]. The NDF and ADF were determined using fiber bags and Fiber Analyzer (Ankom Technology, Macedon, NY, USA). The GE was determined by an Automatic Energy Analyzer (Parr 1281, Moline, IL, USA). Amino acids in diets and digesta were analyzed using the methods described by Zeng et al [20].

The chromium content in the diets and feces was measured using an atomic absorption spectrophotometer (Z-5000; Hitachi, Tokyo, Japan) according to the procedure of Williams et al [22]. Organic matter (OM) was calculated as 1–ash content (DM-base). Nutrient digestibility was determined by the equation as follows:

$$\begin{array}{l} \text{ATTD}=1-({\text{Cr}}_{\text{diet}}\times {\text{nutrient}}_{\text{feces}})/({\text{Cr}}_{\text{feces}}\times {\text{nutrient}}_{\text{diet}})\\ \text{AID}=1-({\text{Cr}}_{\text{diet}}\times {\text{nutrient}}_{\text{digesta}})/({\text{Cr}}_{\text{digesta}}\times {\text{nutrient}}_{\text{diet}}) \end{array}$$

Fresh fecal samples collected on d 28 were used for analy sis of VFAs. Frozen samples were thawed at room temperature and approximately 1.5 g of sample was placed into a centrifuge tube, and mixed with 1.5 mL of sterile water. The content was thoroughly mixed and centrifuged at 15,000×g for 10 minutes at 4°C. The supernatant (1 mL) was transferred into a gas chromatograph sample bottle, and 200 μL meta-phosphoric acid was added. The bottle was immersed in ice for 30 minutes and then centrifuged at 15,000×g for 10 minutes at 4°C. A Hewlett Packard 5890 gas chromatograph (HP, Palo Alto, CA, USA) was used to determine VFA concentrations.

The VFA concentrations of frozen digesta of cecum and colon were determined using a modified method. A 1.0 g sample was diluted with 2.0 mL of 0.10 mol/L HCl solution and put on ice for 30 min and then centrifuged at 12,000×g at 4°C for 15 min. Exactly 1.0 mL of the supernatant was passed through a 0.22 mm Nylon Membrane Filter (Millipore, Bedford, OH, USA) and then 5.0 L of the solution was injected into a gas chromatographic system (Agilent HP 6890 Series, Santa Clara, CA, USA).

Total bacteria genomic DNA extraction was performed from cecal specimens by use of the E.Z.N.A. Stool DNA Kits (Omega Biotek, Norcross, GA, USA). The microbial 16S rDNA was amplified with indexes and adaptors-linked universal primers (341F: ACTCCTACGGGRSGCAGCAG, 806R: GGA CTACVVGGGTATCTAATC) targeting the V3-V4 region, polymerase chain reaction (PCR) was performed using KAPA HiFi Hotstart ReadyMix PCR kit high fidelity enzyme to ensure the accuracy and efficiency of the expansion. The PCR products were detected w 20 g/kg agarose gel electrophoresis, and then recovered by AxyPrep DNA gel Recovery Kit (AXYGEN, San Francisco, CA, USA). Library quality inspection was quantified by Qubit 2.0 Fluorometer (Thermo Fisher Scientific, Waltham, MA, USA) to pool into even concentration, and then sequenced on Illumina HiSeq PE250 platform (Illumina, San Diego, CA, USA) for paired-end reads of 250 bp. The paired-end reads were assembled into longer tags and quality-filtered to remove tags with length of <220 nt, average quality score of <20, and tags containing >3 ambiguous bases by PANDAseq for Clean Reads. All Clean Reads were compared to operational taxonomic unit (OTU) sequences to obtain the final Mapped Reads [23]. After discarding the singletons, the high-quality tags were clustered into OTUs for species taxonomy by using UPARSE with a similarity threshold of 0.97. And the OTUs were further subjected to the taxonomy-based analysis with the ribosomal database project (RDP) database, using the RDP classifier at a 0.80 confidence level. A heat map was created using R. Cluster analysis was done with Cluster 3.0 (Sun Microsystems, Inc., Santa Clara, CA, USA). Alpha diversity (Simpson and Shannon) and beta diversity were analyzed using QIIME (University of California, San Diego, CA, USA). Linear discriminant analyses and effect size (LEfSe) analyses were performed with the LEfSe tool [24]. The relative abundance of bacteria was expressed as the percentage.

### Statistical analysis

All data were analyzed using the Proc-Mixed procedure of SAS (SAS Inst. Inc., Cary, NC, USA). The statistical model for all the values had treatment and period as fixed effects and pig as a random effect. Statistical differences among treatments were separated by Student-Neuman-Keul’s multiple range tests. Values were presented as least square means with standard error of the mean. Statistically significant differences were declared at p<0.05, and differences at 0.05≤p<0.10 were considered a trend toward significance.

## RESULTS

### Nutrients digestibility

Compared to CTR, pigs fed diets supplemented with MOA tended to increase (p = 0.08) the AID of DM. The ATTD of GE, DM, OM was improved (p<0.05) in pigs supplemented with diets containing MOA, while ATTD of NDF was improved (p<0.05) in pigs fed a diet supplemented with MOA2 compared to CTR (Table 2). There was no significant difference between the CTR diet and the MOA diet for the AID of amino acids (Table 3).

### Total volatile fatty acid concentration

Compared to CTR, the pH value in ileum was decreased (p< 0.05) in pigs fed diets supplemented with MOA (MOA1 or MOA2). The concentrations of lactic acid and TVFA in ileum were improved (p<0.01) in pigs supplemented with diets containing MOA compared to CTR (Table 4). In feces, pigs fed diets supplemented with MOA showed higher (p<0.05) concentration of acetic acid and a tendency (p = 0.06) for an improved content of lactic acid, while these pigs had lower (p<0.05) content of formic acid. The concentration of TVFA in feces was increased (p<0.05) in diets supplemented with of MOA1 of pigs, while pigs fed diet containing MOA1 showed higher (p<0.05) concentration of butyric acid in feces compared to CTR (Table 5).

### Intestinal microbiota

**Figure 1:**
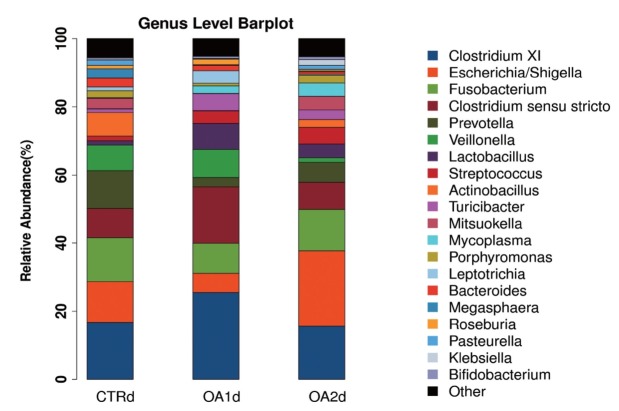
Genus-level distribution of bacteria in the feces of growing-finishing pigs fed basal diet (CTR), basal diet with 3,000 g/kg mixed organic acid 1 (MOA1), or basal diet with 2,000 g/kg mixed organic acid 2 (MOA2).

**Figure 2:**
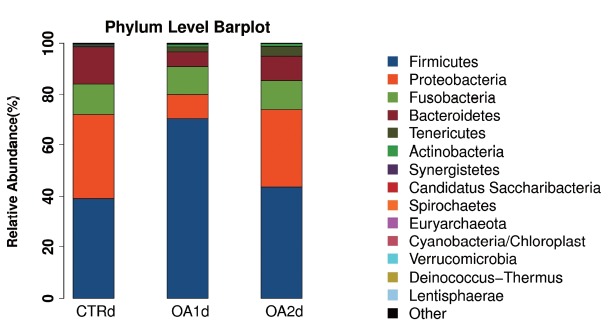
Phylum-level distribution of bacteria in the feces of growing-finishing pigs fed basal diet (CTR), basal diet with 3,000 g/kg mixed organic acid 1 (MOA1), or basal diet with 2,000 g/kg mixed organic acid 2 (MOA2).

**Figure 3:**
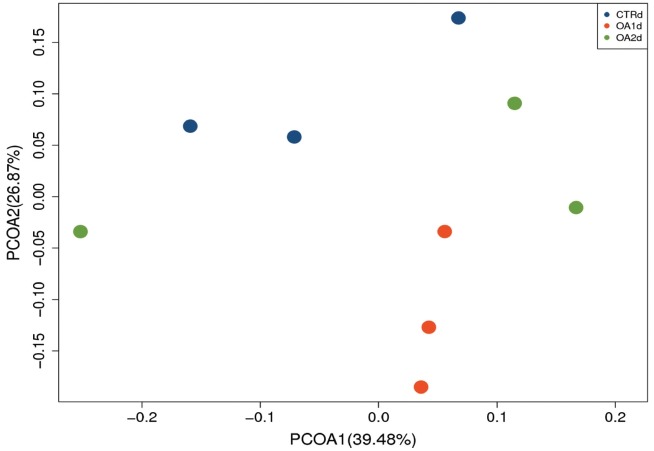
Principal coordinates analysis (PCoA) of the bacterial diversity based on the weighted UniFrac distances between the microbiome profiles of pigs fed basal diet (CTR), basal diet with 3,000 g/kg mixed organic acid 1 (MOA1), or basal diet with 2,000 g/kg mixed organic acid 2 (MOA2). n = 3 per treatment; blue for CTR, red for MOA1, green for MOA2.

No significant differences of the intestinal microbiota concentration were observed in feces of pigs fed CTR and MOA. But a noticeable increase was observed in *Lactobacillus* by the MOA1 treatment, from 1.3% in the CTR pigs to 7.6% in the MOA1 pigs (data not shown). The decreases in *Escherichia* abundance with exposure to the MOA1 were significant: from 12.0% in the feces of the CTR pigs to 5.7% with the MOA1 treatment (Figure 1). An obvious increase was observed in *Firmucutes* by the MOA1 treatment, from 39.1% in the CTR pigs to 70.4% in the MOA1 pigs (Figure 2). A noticeable decrease was observed in *Bacteroidetes* by the MOA1 treatment, from 14.5% in the CTR pigs to 5.7% in the MOA1 pigs (data not shown). For the *Firmicutes*/*Bacteroidetes* ratio, we observed significant differences between CTR (2.7) and MOA1 (12.4). There is a clear distinction between CTR, MOA1, and MOA2 (Figure 3).

## DISCUSSION

Dietary acidification with OA has been shown to improve the performance of weaned piglets and growing-finishing pigs. Previous research reported that dietary supplementation with a combination of formic acid, citric acid, and benzoic acid could improve ADG and feed conversion ratio in growing pigs, which was mainly due to the improved ATTD of DM and GE by MOA [7]. In the present study, the ATTD of GE, DM, and OM were significantly improved in pigs fed with MOA. Meanwhile, we found that there were no significant differences between the AID of GE, CP, and OM in ileum of pigs fed CTR and MOA except MOA had a tendency to increase AID of DM compared to CTR. Our results show that the nutrients digestibility in ileum and feces are coincident, which indicates that OA might be functional mainly in large intestine. Therefore, the mechanism of the MOA increase in the digestibility of nutrients might not be from the beneficial effects of lowing gastric pH and increasing secretion of digestive enzymes in pigs, which is inconsistent with the conjecture of Tonel [25]. In pigs, most nutrients (protein, carbohydrates, and fat) are absorbed in the small intestine by endogenous digestion. Feed components undigested in the small intestine (mainly dietary fiber) are fermented by micro-organisms in the large intestine and the products of this fermentation are partly absorbed by the host animal [15]. In our study, the ATTD of NDF in pigs fed the MOA2 diet was higher than CTR, which might be due to MOA encouraging migration of fiber fermenting bacteria in the end of small intestine and creating a better environment for bacteria that ferment NSP, therefore improving ATTD of crude fiber, total carbohydrates and NSP [26].

Currently, our research also showed there were no signifi cant differences on AID of amino acids between pigs fed diets with MOA and CTR. Several studies have demonstrated that dietary OA supplementation has a positive effect on the AID of CP and amino acids in growing-finishing pigs [8,27], which is inconsistent with our study. A possible reason for this variable effect may be the difference in dietary buffering capacity and combination of feed ingredients.

The SCFA, produced by microbial fermentation of carbo hydrates in the large intestine, have abilities to provide enough energy for pigs (e.g. butyric acid) and protect piglets against pathogenic microorganisms, therefore maintaining the intestinal health. Among all the SCFA, butyric acid, produced by fermentation of carbohydrates and NSP in large intestine, has a positive effect on epithelial cell growth, blood flow, and absorptive functions in pigs [28]. In the current study, dietary MOA supplementation resulted in a significantly higher TVFA content in ileum, which is related to the higher ATTD of NDF in pigs fed MOA2. This relationship indicated MOA might help to promote some specific microbes proliferate rapidly and utilize carbohydrates in large intestine to produce SCFA, which is also shown in previous result where a combination of 5,000 mg/kg formic acid and essential oil significantly increased the ATTD of NSP and total carbohydrates [26]. However, these specific microbes remain to be investigated. Dietary MOA1 and MOA2 supplementation in growing-finishing pigs could also increase the content of TVFA in the ileum of the finishing piglets, which indicated that MOA1 and MOA2 also improved the metabolism of intestinal microbes in ileum in growing-finishing pigs. The increased lactic acid concentration in ileum of pigs in our study was inconsistent with previous studies, which showed the dietary MOA could decrease the lactic acid concentration [8]. The possible reason could be the difference among dietary carbohydrate composition, which could influence the microbial fermentation and nutrient competition between bacteria and host.

Under the action of MOA1, the content of acetic acid and butyric acid in feces was significantly increased, and the content of TVFA was also improved in the present study. This may be due to the MOA promoting the use of microbial fiber in this experiment, which could promote the higher digestibility of NDF, similar findings also showed formic acid in MOA could significantly increases the apparent fecal digestibility of crude fiber, carbohydrate and NSP [27], while our study also showed this tendency, which is possibly due to the reduction of *E. coli* in the distal part of the gastrointestinal tract to provide a better environment for NSP fermentative bacteria by MOA. There were significant differences between the TVFA concentration in feces of pigs fed MOA1 and MOA2, which means that MOA2 didn’t increase fiber fermentation in the large intestine significantly compared with MOA1. The possible reason is that MOA2 mainly consist of MCFA, which are readily absorbed in the stomach or in the upper digestive tract [29].

Same as weaned piglets, digestive disorder is also a severe problem in growing-finishing pigs. When pigs from different rearing compartments are fed a different diet in a uniform fattening unit, the number and species of intestinal micro-organisms change dramatically. Study showed that dietary supplementation with OA can reduce the proliferation of pathogenic bacteria such as *E. coli*, which may due to the dietary OA maintain a lower pH in the gastrointestinal tract [30]. Besides, an inhibitory effect was observed on the general population of pathogenic bacteria throughout the gastrointestinal tract in pigs fed diets containing a combination of organic acids and medium-chain fatty acids [10]. Similar findings showed that formic, benzoic and sorbic acids have a strong antimicrobial action against gut microbial pathogens respectively [31,32]. The antimicrobial mechanism of MOA is their ability to enter bacterium, depolarize its membrane, and then influence its nutrient metabolism [33]. Besides, microbial growth in the large intestine also is affected by the change of pH in the stomach and later in the small intestine [15].

In our study, the microbiota in feces of pigs fed MOA1 shows a higher proportion of *Firmicutes* and a lower population of *Bacteroides* compared to CTR, which increases the *Firmicutes*/*Bacteroidetes* ratio. It has been shown that a decreased *Firmicutes*/*Bacteroidetes* ratio is directly related to weight loss [34]. Therefore, this indicated that the supplementation of MOA1 has a beneficial effect on growth performance by modulating the microbial population. As shown in our study, a visible increase in *Lactobacillus* abundance was observed in the feces of pigs fed MOA1 diet compared with CTR, which indicated favorable modulatory effect of MOA1 on large intestine microbial population. In addition, compared to pigs fed CTR, pigs fed MOA1 had a lower *Escherichia* count in the feces. Studies reported an increased number of *Lactobacilli* and a reduction of *E. coli* in the gut microbiota of pigs fed OA, which is consistent with our research [35]. Previous study also reported that MOA could reduce the amount of total anaerobic bacteria and *E. coli*, which is beneficial for intestinal health of pigs [13]. The inclusion of the MOA1 in the pig diets modified the metabolic activity and composition of hindgut microbiota, which possibly increased the fiber fermentation derived improved nutrient digestibility in the large intestine. These were probably why the TVFA of pigs fed MOA1 was increased in response to the modulated gut microflora. Besides, the carbohydrate composition of feed for pigs is thought to be a determinant of the intestinal flora, which needs to be further investigated.

## CONCLUSION

The present research demonstrated dietary MOA1 or MOA2 supplementation in high fiber diets could improve the ATTD of GE, DM, OM, and NDF. At the same time, dietary MOA supplementation tends to improve the AID of DM, lower pH value, higher concentrations of lactic acid and TVFA in ileum, and improves the concentrations of TVFA in feces. Besides, adding MOA1 to high wheat bran diet for growing-finishing pigs could also increase the concentration of *Lactobacillus* and decreased the concentration of *Escherichia*.

## Figures and Tables

**Table 1:** Ingredients and nutrient contents of diets fed in the experiment (%, as-fed basis)

Items	
Ingredients	
Corn	54.43
Soybean meal (43%)	6.30
Wheat bran	30.00
Soy oil	6.33
Calcium hydrogen phosphate	0.03
Limestone	1.18
Salt	0.30
L-lysine HCl (78%)	0.47
DL-methionine (98%)	0.05
L-threonine (98%)	0.14
L-tryptophan (98%)	0.02
Chromic oxide	0.25
Vitamin-mineral premix1)	0.50
Nutrient composition2)	
Digestible energy (MJ/kg)	14.23
Crude protein	12.77
Calcium	0.52
Digestible phosphorus	0.14
Lysine	0.87
Methionine	0.50
Threonine	0.46
Tryptophan	0.13

1) Vitamin and mineral premix provided the following per kilogram of diet: Mn, 50 mg (MnO); Fe, 125 mg (FeSO_4_.H_2_O); Zn, 125 mg (ZnO); Cu, 150 mg (CuSO_4_.5H_2_O); I, 50 mg (CaI_2_); Se, 0.30 mg (Na_2_SeO_3_); retinyl acetate, 4,500 IU; cholecalciferol, 1,350 IU; DL-α-tocopheryl acetate, 13.5 mg; menadione sodium bisulphite complex, 2.7 mg; niacin, 18 mg; vitamin B_12_, 27.6 μg; thiamine, 0.6 mg; pyridoxine, 0.9 mg; riboflavin, 1.8 mg; D-Ca-pantothenate, 10.8 mg; nicotinic acid, 30.3 mg.

2) The digestible energy, calcium and digestible phosphorus mean calculated values, the rest are analyzed values.

**Table 2:** Effects of mixed organic acids on nutrients digestibility of growing-finishing pigs (%)

Item	CTR1)	MOA11)	MOA21)	SEM	p-value
Ileum					
Gross energy	63.34	66.62	68.24	1.84	0.27
Dry matter	59.00^d^	65.61^c^	69.53^c^	2.40	0.08
Crude protein	77.19	73.74	75.06	3.01	0.88
Organic matter	63.96	67.88	67.86	1.74	0.29
Feces					
Gross energy	80.34^b^	82.81^a^	83.54^a^	0.37	<0.01
Dry matter	79.24^b^	81.01^a^	81.80^a^	0.37	0.02
Organic matter	81.88^b^	83.64^a^	84.20^a^	0.43	0.04
Neutral detergent fiber	51.75^b^	50.95^b^	58.56^a^	0.70	<0.01
Acid detergent fiber	44.61	49.78	48.45	1.40	0.12

SEM, standard error of the mean.

1) CTR, the control; MOA1, mixed organic acid 1; MOA2, mixed organic acid 2.

a–b Different superscripts within a row indicate a significant difference (p≤0.05).

c–d Different superscripts within a row indicate a tendency of difference (0.05<p≤0.10).

**Table 3:** Effects of mixed organic acids on apparent ileum digestibility of amino growing-finishing pigs (%)

Item	CTR1)	MOA11)	MOA21)	SEM	p-value
Aspartic acid	79.03	76.00	82.62	2.51	0.25
Threonine	76.68	75.19	79.63	2.72	0.54
Serine	80.04	76.70	82.23	2.96	0.46
Glutamate	87.63	86.48	89.68	1.20	0.24
Proline	77.23	81.22	85.54	4.81	0.51
Glycine	65.17	61.43	67.54	5.42	0.36
Alanine	76.26	74.23	79.94	2.43	0.37
Cystine	88.18	85.01	87.72	5.58	0.34
Valine	77.05	77.08	86.74	2.68	0.57
Methionine	79.71	76.42	83.16	1.51	0.12
Isoleucine	83.18	80.75	86.40	7.15	0.22
Leucine	74.37	75.00	78.86	1.94	0.61
Tyrosine	81.92	79.63	84.29	2.01	0.26
Phenylalanine	89.75	88.86	91.21	3.33	0.12
Lysine	70.01	63.99	72.43	1.77	0.39
Ammonia	85.37	81.89	86.88	0.67	0.21

SEM, standard error of the mean.

1) CTR, the control; MOA1, mixed organic acid 1; MOA2, mixed organic acid 2.

**Table 4:** Effects of mixed organic acids on the volatile fatty acids in the ileum of growing-finishing pigs

Item	CTR1)	MOA11)	MOA21)	SEM	p-value
pH value	7.32^a^	6.80^b^	6.65^b^	0.12	0.01
Formic acid (mg/g)	0.34	0.40	0.42	0.10	0.86
Acetic acid (mg/g)	0.42	0.45	0.64	0.06	0.12
Propionic acid (mg/g)	0.05	0.05	0.04	0.02	0.86
Lactic acid (mg/g)	0.23^c^	2.16^a^	1.30^b^	0.13	<0.01
Isobutyric acid (mg/g)	1.05^b^	3.06^a^	2.40^a^	0.22	<0.01
Total volatile fatty acid (mg/g)	2.09^b^	6.12^a^	4.80^a^	0.45	<0.01

SEM, standard error of the mean.

1) CTR, the control; MOA1, mixed organic acid 1; MOA2, mixed organic acid 2.

a–c Different superscripts within a row indicate a significant difference (p≤0.05).

**Table 5:** Effects of mixed organic acids on the volatile fatty acids in feces of growing-finishing pigs (mg/g)

Item	CTR1)	MOA11)	MOA21)	SEM	p-value
Formic acid	0.14^a^	0.05^b^	0.05^b^	0.01	<0.01
Acetic acid	2.28^b^	3.50^a^	3.03^a^	0.16	0.01
Propionic acid	1.02	1.58	1.40	0.22	0.29
Lactic acid	0.06^d^	0.15^c^	0.29^c^	0.05	0.06
Butyric acid	0.53^b^	1.18^a^	0.68^b^	0.07	<0.01
Isobutyric acid	0.13	0.17	0.09	0.03	0.33
Valeric acid	0.17	0.25	0.22	0.06	0.60
Isovaleric acid	0.09	0.15	0.08	0.03	0.24
Total volatile fatty acid	4.41^b^	7.03^a^	5.84^ab^	0.44	0.03

SEM, standard error of the mean.

1) CTR, the control; MOA1, mixed organic acid 1; MOA2, mixed organic acid 2.

a–b Different superscripts within a row indicate a significant difference (p≤0.05).

c–d Different superscripts within a row indicate a tendency of difference (0.05<p≤0.10).
